# Activating Killer Cell Ig-Like Receptors in Health and Disease

**DOI:** 10.3389/fimmu.2014.00184

**Published:** 2014-04-22

**Authors:** Martin A. Ivarsson, Jakob Michaëlsson, Cyril Fauriat

**Affiliations:** ^1^Center for Infectious Medicine, Karolinska Institutet, Karolinska University Hospital Huddinge, Stockholm, Sweden; ^2^U1068, CRCM, Immunity and Cancer, INSERM, Marseille, France; ^3^Institut Paoli-Calmettes, Marseille, France; ^4^UM 105, Aix-Marseille Université, Marseille, France; ^5^UMR 7258, CNRS, Marseille, France; ^6^U1068, CRCM, Plateforme d’Immunomonitoring en Cancérologie, INSERM, Marseille, France

**Keywords:** NK cells, KIR, HLA, education, infection diseases, cancer, pregnancy, autoimmune diseases

## Abstract

Expression of non-rearranged HLA class I-binding receptors characterizes human and mouse NK cells. The postulation of the missing-self hypothesis some 30 years ago triggered the subsequent search and discovery of inhibitory MHC-receptors, both in humans and mice. These receptors have two functions: (i) to control the threshold for NK cell activation, a process termed “licensing” or “education,” and (ii) to inhibit NK cell activation during interactions with healthy HLA class I-expressing cells. The discovery of activating forms of KIRs (aKIR) challenged the concept of NK cell tolerance in steady state, as well as during immune challenge: what is the biological role of the activating KIR, in particular when NK cells express aKIRs in the absence of inhibitory receptors? Recently it was shown that aKIRs also participate in the education of NK cells. However, instead of lowering the threshold of activation like iKIRs, the expression of aKIRs has the opposite effect, i.e., rendering NK cells hyporesponsive. These findings may have consequences during NK cell response to viral infection, in cancer development, and in the initial stages of pregnancy. Here we review the current knowledge of activating KIRs, including the biological concept of aKIR-dependent NK cell education, and their impact in health and disease.

## Introduction

The history of non-rearranged MHC class I-binding receptors has been a passionate story for three decades. Shortly after the discovery of NK cells in the mid-1970s (1, 2) MHC class I was suspected to be important for regulating NK cell responses (3–5). The subsequent postulation of the “missing-self” hypothesis (1, 2, 6), predicted that NK cells would express inhibitory MHC class I-binding receptors. In 1992, the discovery of inhibitory MHC class I-binding Ly49 receptors in mice transformed the “missing-self” hypothesis into a now well-admitted dogma (3–5, 7). Shortly after, human inhibitory receptors binding to HLA-C (KIR2DL1/P58.1/EB6 and KIR2DL3/P58.2/GL183) (8–10) and HLA-B (NKB1/KIR3DL1) (11), were identified as functional analogs to the murine inhibitory Ly49 receptors. Moretta and Colleagues later discovered that the monoclonal antibody clone EB6 also could recognize a second receptor p50 (KIR2DS1), and that this receptor transmitted activating signals upon recognition of HLA-Cw4 targets (12). The genes coding for these receptors were later cloned and were confirmed to be specific for HLA-B and -C molecules (13). Since the initial discovery of the first inhibitory and activating KIRs in humans (referred to iKIRs and aKIRs respectively in the rest of the manuscript), a number of additional KIRs have been identified. The KIR family now includes seven iKIR and six aKIR, in addition to KIR2DL4, which has both inhibitory and activating function. Adding to the complexity, each KIR gene is highly polymorphic, and the product of different alleles can interact more or less strongly with different HLA class I alleles (Table 1).

**Table 1:** **Activating and inhibitory KIRs and ligands**.

Activating KIR	Ligand	Detectable by FACS	Reference
2DS1	HLA-C2 (weaker than 2DL1)	Yes	(14, 15)
2DS2	HLA-C1 (weak), HLA-A*11:01	Yes	(16–18)
2DS3	Unknown	? (Retained intracellularly?)	(19)
2DS4	HLA-C*05:01, A*11:02, C*16:01	Yes	(20)
2DS5	Unknown	Yes	(21–23)
3DS1	Unknown	Yes	(24)

**Inhibitory KIR**	**Ligand**	**Detectable by FACS**	

2DL1	HLA-C2 (N77/K80)	Yes	(8)
2DL2	HLA-C1 (S77/N80), HLA-C2, HLA-B*46:01 and HLA-B*73:01 (C1 epitope)	Yes	(9, 25)
2DL3	HLA-C1 (S77/N80), HLA-C2, HLA-B*46:01 and HLA-B*73:01 (C1 epitope)	Yes	(9, 25)
2DL4	HLA-G (intracellular interaction?)	Yes (not shown *in vivo*)	(26)
2DL5A/B	Unknown	Yes	(27)
3DL1	HLA-A (with Bw4 motif), HLA-Bw4	Yes	(11, 28)
3DL2	HLA-A3/A11	Yes	(29, 30)
3DL3	Unknown	? (Methylated promoter)	(31)

All KIR genes are encoded in the leukocyte receptor complex (LRC) in the chromosome 19 (19q14.3). Thus, the LRC includes the 15 genes of the KIR family. Importantly, for some KIRs, there is extreme allele variability, whereas some KIR genes are highly conserved and exist only as few alleles. The variability of KIR alleles can be nicely visualized^1^. Interestingly, compared to iKIR, aKIR display rather limited allele variability compared to iKIR. For instance there are more than 75 alleles coding for KIR3DL1 and only 17 coding for KIR3DS1 (32), and *KIR2DS1* gene encompassed only 16 alleles in contrast to *KIR2DL1* gene, which include more than 40 alleles^2^.

In addition to allele polymorphism, there is strong haplotype variability due to the number of KIR genes present in the LRC. Two types of KIR haplotypes, A and B, have been defined pending on gene content. Group B haplotypes are defined by the presence of at least one of the following KIR genes: *KIR2DL5, KIR2DS1, KIR2DS2, KIR2DS3, KIR2DS5*, and *KIR3DS1*. Conversely, group A haplotypes are characterized by the absence of all these genes. All information about KIR alleles and haplotypes has been collected in the KIR web-based database (see text footnote 1). In addition, another repository has been created for association between KIR and diseases^3^ (33).

Currently, the aKIR include KIR2DS1, KIR2DS2, KIR2DS3, KIR2DS4, KIR2DS5, and KIR3DS1 (Table 1). The specificity of each receptor as well as their cell surface expression has been elusive for many years, despite the fact that the extracellular domains of these molecules are extremely similar to their inhibitory counterparts, in terms of sequence and 3D structure. Studies implicating aKIRs in diseases are therefore almost exclusively based on genetic studies. More recently, new staining protocols and monoclonal antibodies have allowed identification of cells expressing KIR2DS1, KIR2DS2, KIR3DS1, and KIR2DS5 (15, 16, 22–24, 34–38). The possibility to identify the specific expression of distinct aKIRs has made it possible to determine how these receptors regulate NK cell function at the single level, and in concert with other activating and inhibitory HLA class I-binding receptors.

KIR2DL4 remains a particular KIR because of its structure, expression, and functions (39). Hence, this receptor is expressed by all NK cells, in contrast to other KIRs, which are clonally expressed. *KIR2DL4* has also a lower diversity but its allele diversity influences the surface expression and function in NK cells (40, 41). Hence, the 9A allele of KIR2DL4 is not stable at the plasma membrane and is rapidly recycled. Consequently, intracellular KIR2DL4, engagement with its ligand HLA-G in endocytosis vesicles resulted in production of cytokines such as IFN-γ but also other cytokines such as IL-1β, TNF-α, and IL-8, etc., [nicely reviewed by Rajagopalan and Long (39)].

## The Elusive Nature of Activating KIR Ligands

Despite the high degree of sequence homology between activating and inhibitory KIRs, the specificities of most aKIRs remain elusive. In particular, this is true for KIR2DS1, KIR2DS2, and KIR3DS1. KIR2DS1, the first described, and most well-studied activating KIR, binds to HLA-C molecules within the C2 group (with the N77/K80 motif) (12). KIR2DS2 has been shown to recognize group 1 HLA-C and induce a KIR–HLA-dependent NK cell activation (42, 43). However, many other studies have failed to identify KIR2DS2–HLA-C1 interaction [nicely reviewed by Moesta and Parham (44)]. Unexpectedly, a recent publication revealed that KIR2DS2 recognizes HLA-A*11 (18). With respect to KIR3DS1, while there are indications that KIR3DS1 interacts with HLA-Bw4^80I^, there have been no studies demonstrating a direct interaction between these two molecules (24). In contrast to the above-mentioned aKIRs, KIR2DS3 and KIR2DS5 have no inhibitory counterparts. Although detection at the cell surface of NK cells has been recently proven possible (19, 21), the identification of their respective ligand(s) has not been successful. KIR2DS4 however has been shown to interact with HLA-A*11:02, HLA-C*05:01, and HLA-C*16:01 (20).

It is possible that the aKIRs have HLA class I as their major ligands. However, it also remains possible that these receptors could recognize altered HLA class I complexes, e.g. specific HLA/peptide complexes, or complexes of HLA class I together with viral proteins, or non-HLA class I ligands altogether (45). For example, KIR2DS4 has been suggested to bind to an unidentified protein expressed on melanoma-derived tumor cells, independently of HLA class I (46). In mice, the activating Ly49H receptor directly recognizes the CMV-encoded protein m157 (47, 48), and thereby allows NK cells to control CMV infection. Similarly, mouse CMV m04 together with the MHC class I molecule H-2D^k^ allows recognition of CMV-infected cells by NK cells expressing the activating receptor Ly49P (49). It thus remains possible that the aKIRs could directly, or indirectly, recognize virally encoded ligands, although such ligands have yet to be identified in humans.

Several iKIRs display a certain degree of peptide selectivity, where the nature of the HLA class I presented peptide affects the binding to KIRs (43, 45, 50, 51). Similarly, KIR2DS1 displays a certain degree of peptide selectivity in its binding to HLA-Cw4 (43), indicating that also aKIRs can be modulated by the nature of the presented peptide. Noteworthy the recent crystal structure of KIR2DS2–HLA-A*11:01 was obtained in complex with a vaccinia viral peptide (18). Moreover, the authors showed that peptide sequence affects the binding of KIR–HLA. Additionally, HLA allele themselves can display a different affinity for their attributed KIR (42).

In addition to alterations in peptide content during, e.g., viral infections, the overall cell surface pattern of MHC class I can be altered. Interestingly, it was recently shown in mouse, that peptide-specific clusters of HLA class I/peptide complexes are formed on the cell surface of infected cells, depending on the nature of the peptide presented (52). These peptide-specific clusters create a high level of expression of a given MHC class I/peptide complex on the cell surface of infected cells, which allow a more efficient recognition by cognate T cell receptors (52). It is tempting to speculate that in humans, the clustering of specific HLA class I/peptide complexes during infections could be one way for aKIRs to recognize virus-infected cells. Noteworthy, most studies on aKIRs have evidenced a weaker affinity for their ligands compared to their inhibitory counterparts (43, 44).

## Education of NK Cells by Activating KIR

Activating KIRs convey their signals through the adaptor KARAP/DAP12 (53, 54). Upon ligation with antibodies or ligands, Src kinase-dependent phosphorylation of DAP12 allows the recruitment of SYK and the triggering of LAT-dependent signaling pathways (PI3K–AKT, NKFAT, and MAPK) (55).

As mentioned above, the ligands for most aKIRs remain elusive and KIR2DS1 is the activating receptor with the best described ligand (HLA-C2) (43, 56, 57). Several studies have shown that KIR2DS1^+^ NK cells efficiently kill HLA-Cw4^+^ target cells (12, 14, 15, 43, 45, 58). However, it has been hard to reconcile the fact that NK cells can express activating receptors whose ligand is expressed on healthy cells, as this interaction potentially could lead to autoreactivity. To cope with potential autoreactivity, it is likely that NK cells, much like T cells, have evolved a system for tolerance also for aKIRs. One possibility is negative selection of NK cells expressing self-HLA class I-binding aKIRs in the absence of self-specific inhibitory KIRs. However, recent data refuted this hypothesis, as NK cells expressing KIR2DS1 in the absence of self-specific inhibitory KIRs or CD94/NKG2A can be detected in HLA-C2^+^ donors (15, 36, 37, 59).

An alternative hypothesis is that NK cells expressing aKIRs are not deleted, but rather rendered hyporesponsive if the ligand is present in the host. Indeed, in HLA-C2 homozygous donors NK cells expressing KIR2DS1 in the absence of self-HLA class I-specific KIRs (KIR2DS1sp) were hyporesponsive to target cell stimulation, whereas no such hyporesponsiveness was observed in HLA-C1 homozygous donors (15), indicating that the responsiveness of KIR2DS1^+^ NK cells is tuned down in HLA-C2 homozygous donors (15, 59). Similarly, IL-2 activated KIR2DS1sp NK cell lines and clones from HLA-C2 homozygous donors have been reported to have lower responses to HLA-C2^+^ target cells (14, 59).

This newly identified NK cell education complements education via inhibitory HLA class I-binding receptors (iKIR and NKG2A/CD94), since it renders otherwise potentially autoreactive NK cells self-tolerant. The system of iKIR- and aKIR-mediated education can in some ways be compared to the positive and negative selection of T cells; where T cells are positively selected based on having TCRs that recognize self-MHC class I, and negatively selected if they express a TCR that recognize MHC class I in complex with self-peptides. In comparison with the T cells, education by iKIR would be the analog of positive selection, and education via aKIRs would be the analog of negative selection, with the major difference that T cell selection results in deletion or survival, whereas for NK cells it sets the threshold for activation, without affecting survival (60, 61).

Interestingly, although the mechanisms have not yet been identified, education by aKIRs shares features with the hyporesponsiveness induced by chronic stimulation of other activating receptors expressed by NK cells. For example, chronic exposure to NKG2D ligands in mice renders NK cells hyporesponsive to target cells (62). Similarly, when the ligand (m157) for the activating Ly49H is constitutively expressed, mouse Ly49H^+^ NK cells become hyporesponsive (63). Finally, NK cells in NKp46-deficient mice are more responsive, suggesting that NKp46 hampers the reactivity of NK cells via an unidentified constitutively expressed ligand (64). Thus, the mechanism ensuring tolerance by self-HLA class I-binding aKIRs might be similar to that of these activating receptors. Other examples of activating receptor-dependent NK cell tolerance exist but the molecular mechanisms are likely to be different. For instance, mouse 2B4 may be associated with different adaptors that transmit inhibitory or activating signals (65). Interestingly, the tuning of NK cells expressing self-HLA class I-binding aKIRs seems to be restricted to target cell recognition, as it does not affect the response to cytokine stimulation (15). Furthermore, based on murine studies, education of NK cells appears to be a dynamic and reversible process, both via activating and inhibitory receptors (66, 67). This suggests that NK cells expressing self-HLA class I-binding receptors may be silenced under normal conditions, but once an appropriate signal is given to the cells, they may become an important component of an efficient immune response. For example, sudden changes, in contrast to chronic long-term changes, might provide such a signal, a concept that was recently proposed in the “discontinuity theory for immunity” (68). Compiling all relevant literature, the authors noted that innate immune cells, but also to some extent adaptive immune cells, are tolerant to “self,” irrespective of how aberrant the “self” is, until an “unexpected” event disrupts the equilibrium that is maintaining tolerance, thus awakening the immune effectors.

Overall, the iKIR- and aKIR-dependent education of NK cells by HLA class I molecules, together with the polymorphic nature of KIRs and HLA class I, and the variegated expression of KIRs on NK cells, results in a highly complex system of NK cell regulation. The effects of this complex system are only just starting to be investigated in different diseases, and most available data thus comes from genetic studies.

## Activating KIRs in Viral Infections

Over the past 10 years, a number of studies have aimed at elucidating a potential role of aKIRs in virus infections, including HIV, HCV, and CMV. There is now compelling evidence, although mostly at the genetic level, that aKIRs can affect the outcome of different infections. The large degree of linkage disequilibrium between different *KIR* genes, e.g., *KIR2DL2* and *KIR2DS2*, and *KIR2DS1* and *KIR3DS1*, however makes it hard to pinpoint a specific KIR/HLA class I interaction at the genetic level, as responsible for protection or susceptibility to a given infection. In addition, the lack of well-characterized ligands for several of the aKIRs, e.g., KIR3DS1, KIR2DS2, and KIR2DS3, as well as the lack of specific monoclonal antibodies to these aKIRs have severely hampered investigations of the functional role of these receptors in viral infections. The identification of ligands and the development of new antibodies specific for aKIRs are thus required to move the field forward toward understanding how these receptors regulate NK cell function in different infectious disease settings.

KIR3DS1 is the by far most well-studied aKIR in the setting of infectious diseases [also nicely reviewed by Körner and Altfeld (69)]. The first evidence for a role of KIR3DS1 in viral infections came from genetic studies in HIV-infected individuals where individuals homozygous for *KIR3DS1* progressed slower to AIDS, but only if they also carried *HLA-Bw4* with an isoleucine at position 80 (*HLA-Bw4^80I^*) (70). Interestingly, donors homozygous for *KIR3DS1*, but lacking *HLA-Bw4^80I^* progressed faster to AIDS (70). Furthermore, *HLA-Bw4^80I^*^+^ individuals with increasing number of copies of *KIR3DS1* due to copy number variation have been shown to have lower viral load set points (71). The frequency of *KIR3DS1* positive individuals has also been shown to be higher in HIV-exposed seronegative individuals, compared to HIV seropositive and HIV negative individuals, indicating that KIR3DS1 might also be providing protection against infection (72). In addition, the frequency of KIR3DS1^+^KIR3DL1^−^ NK cells is increased in *HLA-Bw4^80I^*^+^ individuals, both at acute and chronic stages of HIV infection compared to healthy controls (73). The protective effect of KIR3DS1 in HIV infection was further strengthened by evidence that individuals carrying both *KIR3DS1* and *HLA-Bw4^80I^* suppress HIV replication *in vitro* to a greater extent than NK cells from individuals carrying *KIR3DS1* in the absence of *HLA-Bw4^80I^*, or vice versa, carrying *HLA-Bw4^80I^* in the absence of *KIR3DS1* (56). In addition, purified KIR3DS1^+^KIR3DL1^−^ NK cells from *HLA-Bw4^80I^*^+^ individuals suppressed HIV replication *in vitro* to a much greater extent than KIR3DS1^−^KIR3DL1^+^ and KIR3DS1^−^KIR3DL1^−^ NK cells (56), indicating that the effect of KIR3DS1 is likely to be mediated by KIR3DS1^+^ NK cells, and not by KIR3DS1^+^ T cells. The same group subsequently demonstrated that the effect of KIR3DS1 on viral inhibition *in vitro* was highest in individuals carrying *KIR3DS1, HLA-Bw4^80I^*, and at least one copy of *KIR3DL1* (71). Surprisingly, NK cells from *HLA-Bw4^80I^*^+^ individuals homozygous for *KIR3DS1*, but lacking *KIR3DL1*, did not suppress HIV replication *in vitro* to a greater extent than NK cells from *HLA-Bw6* homozygous individuals, indicating that the effect of KIR3DS1 is dependent on both KIR3DL1 and HLA-Bw4^80I^. Interestingly, the expression of KIR3DS1, both at the mRNA and protein level, was elevated in individuals carrying *KIR3DS1* in the presence of two copies of *KIR3DL1*, compared to individuals carrying *KIR3DS1* in the presence of one copy of *KIR3DL1*. However, that study did not compare the expression levels of KIR3DS1 to individuals carrying two copies of *KIR3DS1* in the absence of *KIR3DL1*, making it hard to determine whether increased expression levels of KIR3DS1 alone could explain the increase in viral inhibition, as suggested by the authors. An alternative explanation for the increased suppression of HIV replication by *KIR3DS1/KIR3DL1* heterozygous individuals, compared to *KIR3DS1* homozygous individuals could be an increased efficiency of NK cells co-expressing KIR3DL1 and KIR3DS1, potentially allowing NK cells to sense a down regulation of HLA-Bw4 via KIR3DL1, and at the same time activate them via KIR3DS1-mediated recognition of HLA-Bw4 with, e.g., an altered peptide repertoire. However, sorted KIR3DL1^+^ NK cells from *KIR3DS1/KIR3DL1* heterozygous individuals, that could contain NK cells co-expressing KIR3DL1 and KIR3DS1, did not efficiently suppress HIV replication *in vitro*, compared to NK cells expressing only KIR3DS1 (56). This indicates that co-expression of KIR3DL1 and KIR3DS1 is not required for KIR3DS1-mediated NK cell suppression of HIV replication *in vitro*. It is also noteworthy that very little is known about co-expression of other activating and inhibitory KIRs and other HLA class I-binding receptors (e.g., NKG2A, NKG2C, and LIR-1) on KIR3DS1^+^ NK cells, both in healthy and in HIV-infected individuals.

The complexity of the effects of KIR3DS1 in HIV infection is further highlighted by reports that KIR3DS1 is associated with an increased progression to AIDS, but when restricted to *HLA-Bw4*^+^ individuals, *KIR3DS1* presence is associated with a slower progression (74). Other reports have suggested an HLA-Bw4-independent effect of KIR3DS1 in HIV-infected individuals, at least when measuring CD8 T cell activation, which is strongly associated with HIV disease progression (75). Taken together, the data collected to date indicate a role for KIR3DS1 in HIV disease and progression to AIDS, but it is also clear that more efforts are needed to explain the complex nature of KIR3DS1-mediated protection in HIV-infected individuals, including the identification of a ligand for this receptor.

In contrast to the role of KIR3DS1 in HIV infection, much less is known about the role of other aKIRs in viral infections. Similar to studies of aKIR and HIV, most of the studies have been performed at the genetic level, and commonly in smaller cohorts of patients. Nevertheless, a number of studies have implicated a role for aKIRs in other viral infections. For example, the frequency of *KIR2DS3* positive individuals is higher in patients with chronic HCV infection, compared to individuals that have resolved HCV infection (76). Interestingly, the detrimental effect of *KIR2DS3* was only observed in *HLA-C2*^+^ individuals, which is not believed to be a ligand for KIR2DS3 (76). An increase in *KIR2DS3*, as well as *KIR2DS2* positive individuals has also been reported in a Brazilian cohort of chronically HCV-infected patients (77). Whether the association between *KIR2DS3* and chronic HCV infection reflects a direct interaction between HCV-infected cells and KIR2DS3^+^ NK cell remains unknown since there is neither a known ligand, nor specific antibodies for KIR2DS3. A likely explanation proposed by Dring et al. is that KIR2DS3 is instead a marker of a particular haplotype that is associated with the disease progression (76).

In addition to a role for KIR3DS1 in HIV infection, *KIR2DS2* has been associated with faster progression to AIDS (74). A similar association was found in a West African cohort of HIV-infected women, where haplotype B KIRs, including *KIR2DS2, KIR2DS3*, and *KIR3DS1*, was associated with lower CD4 T cell counts (78). Interestingly, homozygosity for *HLA-C1*, a putative ligand for KIR2DS2 and KIR2DS3, decreased the association between lower CD4 T cell counts and the aKIRs. However, there is no evidence for a direct functional role of KIR2DS2 or KIR2DS3 in recognition of HIV-infected cells. Because HLA-C is not downregulated by HIV-infected cells (79), it is tempting to speculate that NK cells expressing HLA-C-binding aKIRs in the absence of HLA-C-binding iKIRs, could mediate recognition of HIV-infected cells. For example, KIR2DS1^+^KIR2DL1^−^ NK cells, which are activated by interaction with HLA-C2^+^ target cells, could potentially recognize HIV-infected cells in HLA-C2^+^ individuals. The lack of effects of *KIR2DS1/HLA-C2* in HIV infection could potentially be explained by the education of NK cells via aKIRs, where KIR2DS1^+^ NK cells in HLA-C2^+^ individuals are hyporesponsive.

In addition to the associations between activating KIRs and viral infections described above, *KIR2DS1* and *KIR2DS3* have also been associated with fatal outcome in Ebola infection. The frequencies carriers of both these genes were increased in patient cohorts with fatal outcome of Ebola infections, compared to survivors, contacts, and healthy controls (80). However, as the study investigated a rather small cohort, no further dissection of interactions between these aKIRs and HLA-C was possible. Expansions of KIR2DS2^+^, KIR2DS4^+^, and KIR3DS1^+^ NK cells have also been reported to occur in a subset of CMV seropositive individuals (17). However, direct evidence for a role of these aKIRs in the recognition of CMV-infected cells is still lacking.

In summary, although there are a fairly large number of studies associating *KIR* gene content with outcome of viral infections, more direct functional evidence for a role of aKIRs in recognition of virus-infected cells is lacking. The identification of novel, possibly virus-encoded or virus-induced, ligands for activating KIRs, as well as specific monoclonal antibodies, are required to understand the role of these receptors in viral infections.

## Activating KIR in Cancer

A number of studies have investigated the associations between inhibitory and activating KIR and cancer prognosis. However, similar to the role of aKIRs in infections, the evidence for a role for aKIRs in cancer has largely been at the genetic level due to the limitations in available specific monoclonal antibodies. The education of NK cells via aKIR/HLA class I may play a significant role in controlling NK cell responses to transformed cells, as NK cells expressing aKIRs are hyporesponsive in the presence of self-HLA class I ligands. NK cells expressing aKIR in individuals where the ligand is present will be hyporesponsive, and thus may not be able to mount an efficient response against tumor cells in the same individual. Conversely, NK cells expressing aKIRs in individuals where the ligand is not expressed will not be able to recognize the tumor cells due to the lack of the ligand. However, it remains possible that there are hitherto unknown ligands for aKIRs expressed on tumor cells (46), potentially providing a role for aKIRs in various cancers. A recent study evidenced that CCR7 expressed by dendritic cells or lymphoblasts could be transferred to NK cells in a KIR2DS1/HLA-C2-dependent manner. Consequently, CCR7^+^ NK cells would migrate to secondary lymphoid organs and further improve anti-leukemic effect of NK cells, notably after hematopoietic stem cell transplantation (haplo-HSCT) (81).

The role of activating KIRs in cancer seems to vary with type of cancer, therapy and clinical measurement, and evidence for both protective and detrimental effects exist. For example, *KIR2DS1* gene frequency was found to be higher in breast cancer patients compared to healthy controls, and in contrast to *KIR2DL1* was suggested to promote cancer progression (82). Similarly, individuals with chronic myeloid leukemia carrying the *KIR2DS1* gene had a lower response to antibody-treatment with Imatinib (Glivec) (83). In addition, multiple myeloma patients carrying *KIR3DS1* had shorter progression-free survival (PFS), compared to those that lacked *KIR3DS1*, after autologous stem cell transplantation (84). However, among the *KIR3DS1* positive individuals in that study, patients carrying *HLA-Bw4* had longer PFS compared to those lacking *HLA-Bw4*. In contrast, Karabon et al. recently reported that *KIR3DS1* positive individuals with B cell lymphocytic leukemia (B-CLL) had a trend toward longer progression-free survival compared to KIR3DS1 negative individuals. Interestingly however, in the same study co-carriage of *KIR3DS1* among *HLA-Bw4*^+^ patients, as well as co-carriage of *KIR2DS1* in *HLA-C2*^+^ patients were associated with longer PFS (85).

Allogeneic stem cell transplantation to treat leukemia is one setting where NK cells expressing aKIRs may play a significant role, in particular when using KIR ligand mismatched donor/recipient pairs. In this setting, aKIR^+^ NK cells from donors lacking expression of ligands for the aKIRs could potentially recognize the recipients’ leukemia blasts, e.g., when transplanting a *HLA-C2* positive recipient with cells from a *KIR2DS1* positive but *HLA-C2* negative donor. Indeed, it was recently shown in acute myeloid leukemia that patients receiving allografts from *KIR2DS1* positive donors had a lower probability of relapse, but only if the donor was not homozygous for *HLA-C2* (86). Interestingly, there was no difference in relapse between *HLA-C2* homozygous patients receiving a *KIR2DS1* positive allograft, compared to those receiving a *KIR2DS1* negative allograft. In contrast, there was a lower probability of relapse in *HLA-C1/C1* and *HLA-C1/C2* patients receiving a *KIR2DS1* positive allograft, compared to those receiving a *KIR2DS1* negative allograft (86). Based on their data, the authors concluded that it would be beneficial to select *KIR2DS1* positive *HLA-C1* positive donors for transplantation into *HLA-C1* positive donors, and suggested that tolerance-induction via KIR2DS1/HLA-C2 could potentially explain the lack of effects when the allograft was derived from KIR2DS1 positive HLA-C2/C2 donors. The data are also compatible with KIR2DS1^+^ donor NK cell education via interactions with HLA-C2 in the recipient, since there was no effect of donor *KIR2DS1* in *HLA-C2/C2* recipients. However, it is hard to reconcile how KIR2DS1^+^ NK cells could mediate an anti-leukemic effect when transplanted into *HLA-C1* positive donors, as there would be no ligand for KIR2DS1 expressed by the recipient’s leukemia blasts. One possible explanation, which is still compatible with aKIR-mediated education, is that an unknown non-HLA class I ligand is expressed by leukemic blasts, and as such provides a mechanism by which KIR2DS1^+^ NK cells could recognize the leukemia blasts, but only if the donor-derived KIR2DS1^+^ NK cells are not rendered tolerant by the donors or recipient’s HLA-C2. However, no such ligands been identified to date. In addition to the study by Venstrom et al. (86), several other studies have indirectly indicated an effect of activating KIR in hematopoietic cell transplantation. In a study with over 1000 AML patients, receiving grafts from KIR haplotype B donors, in particular KIR cen-B homozygous donors, was associated with a higher relapse-free survival, compared to those receiving grafts from KIR haplotype A/A donors, which lack most aKIRs (87, 88). Interestingly, no effect of donor KIR haplotype B was observed on outcome of ALL in the same study. In contrast to these studies, McQueen et al. reported that KIR haplotype A/A patients with AML/MDS receiving KIR haplotype B grafts (containing aKIR) had a lower survival together with a higher relapse and GvHD rates, compared to KIR haplotype A/A donors receiving a KIR haplotype A/A graft (89). It should however be noted that the number of patients with KIR haplotype A/A receiving a KIR haplotype B graft in that study was low (8–11 patients), making it hard to draw firm conclusions.

More direct evidence for a role of aKIRs in tumor recognition was provided by Pende et al. in patients receiving haploidentical haplo-HSCT from KIR ligand-mismatched donors. Donor-derived KIR2DS1^+^ NK cells were shown to efficiently kill HLA-C2^+^ leukemia blasts, indicating that aKIRs could mediate important anti-leukemic effects in the setting of bone marrow transplantation (90).

Overall, although a number of studies have analyzed the co-carriage of *KIR* genes and their ligands, general conclusions regarding the role of aKIR in cancer and cancer therapy cannot be drawn, as it is likely that the role of aKIRs will vary depending on the type of cancer and treatment. Given the linkage disequilibrium of activating KIRs, large cohorts of well-defined patients are needed to pinpoint the role of specific aKIRs, as well as the presence of their ligands. In addition, functional data investigating the role of aKIRs, and education via these receptors, is largely lacking. More studies monitoring aKIR-dependent NK cell functions are thus warranted in order to decipher the impact of aKIR-mediated education on cancer incidence and progression.

## Activating KIR in Human Reproduction

More and more studies reveal the presence of NK cells in tissue and in particular uterine NK (uNK) cells have been found highly interesting with regard to their regulation by aKIRs. uNK cells represent the dominant leukocyte subset in the decidua (uterine endometrium in pregnancy) during the initial period of pregnancy (91). The uNK cells have been linked to the particular physiological changes that the implantation of the embryo to the uterine wall entails [elegantly reviewed by Mofett and Loke (92)]. Briefly, during the initial stages of pregnancy, fetal extravillous trophoblasts (EVT) implant the embryo by invading into the maternal uterine wall and remodel uterine spiral arteries to ensure the supply of oxygen and nutrients to the growing placenta and fetus. EVTs are peculiar in that they express no HLA-A and -B, but do express HLA-C, -E, and -G. Interestingly, KIR2DL1/S1 and KIR2DL2/3/S2 expression is more frequent on uNK cells than on matched peripheral blood NK cells (93), suggesting a tissue-specific role for KIRs on the uNK cells (which also express KIR2DL4, NKG2A, and LILRB1). The invading EVT reside in the same anatomical location as uNK cells and it has been hypothesized that KIR/KIR ligand combinations thus affect implantation and pregnancy success. In support of this, mothers lacking activating *KIR genes* (haplotype A/A), who consequently express only inhibitory KIR2DL1, in combination with a HLA-C2^+^ fetus, were at higher risk for preeclampsia and IUGR (94). In contrast, presence of the activating *KIR2DS1* gene was protective, which indicated that activation of uNK cells via aKIR might be important in the interaction with EVT. Indeed, the same group recently showed that KIR2DS1 is functional on uNK cells (95). KIR2DS1 had an educating effect on uNK cells in HLA-C2 donors with regard to target cell induced degranulation, similar to the effect described for peripheral blood NK cells (15). However, signaling via KIR2DS1 on uNK cells also led to production of GM-CSF that could be used to attract trophoblasts *in vitro*. Together this provides mechanistic insight into the *in vivo* importance for activating KIR on uNK cells, in their potential interaction with EVT. Regarding other aKIRs on uNK cells, KIR2DS4 is also expressed on uNK cells, and using clonally expanded uNK cells, KIR2DS4 has been shown also to be functional (96). Future experiments will reveal whether KIR2DS4 plays a similar role as KIR2DS1 in the regulation of uNK cell function. HLA-G is expressed by trophoblasts and KIR2DL4 is also expressed by uNK cells. Expression of soluble HLA-G during pregnancy has been documented (92) and correlates with successful pregnancy (97). Consequently, intracellular KIR2DL4, engagement with its ligand HLA-G in endocytosis vesicles would result in production of various cytokines, which may have vascular remodeling consequences similar to KIR2DS1 activation (98).

## Activating KIR in Autoimmune of Inflammatory Disease

The relationships between KIR expression and autoimmune or inflammatory disorders remain largely unknown. Similarly to other diseases, the role of interaction between aKIRs and HLA class I has only been extrapolated from genetic studies looking both at *KIR* and *HLA* gene expression. *KIR2DS1* gene was suggested to be less common in patients with atopic dermatitis compared to healthy controls (99), suggesting that that KIR2DS1 might have a protective effect. In contrast, *KIR2DS1* was found more often in patients with systemic lupus erythematous (100), and the same was true for *KIR3DS1* in multiple sclerosis (MS) (101). Psoriasis vulgaris is also characterized by a clear association between *KIR2DS1* and HLA-Cw6, whose co-carriage was over-represented in patients (102, 103). In this setting, Łuszczek et al. hypothesized that interactions between KIR2DS1 and its ligand would contribute to pathogenesis. Given that KIR2DS1^+^ NK cells are normally hyporesponsive in HLA-C2 positive donors, these studies indicate that there is a potential break of tolerance leading to psoriatic lesions. In contrast, a former study on psoriatic arthritis showed that *KIR2DS1* and *KIR2DS2* were associated with susceptibility to disease, but only in the absence of their cognate HLA ligands (104). Although responsiveness of KIR2DS1^+^ NK cells must be assessed, it is possible that an aKIRs education-dependent higher responsiveness would render KIR2DS1^+^ NK cells able to mediate autoimmune reactions, in the absence of HLA-C2, thus recognizing an unknown ligand.

## Concluding Remarks

Although significant progress has been made in the understanding of aKIRs and their interactions with HLA class I, the role of aKIRs in disease and health still remains largely unexplored. The studies of aKIR/HLA class I associations indicate that aKIRs are likely to play a role in a number of different diseases, including infectious diseases, cancer, and autoimmunity. However, the lack of specific monoclonal antibodies to analyze expression patterns of aKIRs, together with their polymorphic nature and linkage disequilibrium with iKIRs have hampered detailed studies of aKIRs both in disease and in health. The development of specific antibodies, as well as the identification of ligands for aKIRs, potentially including non-HLA class I molecules, would greatly advance our understanding of aKIR biology. Finally, it is important to keep in mind that KIR expression is not restricted to NK cells but expression has also been documented on αβ and γδ T cells (105, 106). Particularly for αβ T cells and infection or autoimmune diseases where polymorphism of HLA is important, the expression of aKIR might shed light on these genetic associations.

## Conflict of Interest Statement

The authors declare that the research was conducted in the absence of any commercial or financial relationships that could be construed as a potential conflict of interest.

## Footnotes

^1^http://www.allelefrequencies.net/kir6008a.asp

^2^http://www.ebi.ac.uk/ipd/kir/

^3^http://www.allelefrequencies.net/diseases/default.asp
